# Construction of a Prognostic Model Using RNA Processing Factor Genes and the Key Role of *NSUN6* in Glioma Outcomes

**DOI:** 10.1111/jcmm.70668

**Published:** 2025-06-25

**Authors:** Jiarui Chen, Caidi Ying, Zhaowen Gu, Bingrui Zhu, Junjie Wang, Yajun Qian, Haiyan Zheng, Jianming Zhang, Yongjie Wang

**Affiliations:** ^1^ The Second Affiliated Hospital of Zhejiang University School of Medicine Hangzhou China; ^2^ Department of Neurosurgery, 4th Affiliated Hospital, School of Medicine Zhejiang University Zhejiang China; ^3^ Clinical Research Center for Neurological Diseases of Zhejiang Province Hangzhou China; ^4^ Key Laboratory of Precise Treatment and Clinical Translational Research of Neurological Diseases Zhejiang Hangzhou China

**Keywords:** glioma, *NSUN6*, prognostic model, RNA processing factors, survival analysis

## Abstract

Glioma is the most common malignant brain tumor and remains associated with a poor prognosis and limited predictive tools. The dysregulation of RNA processing factor genes has been implicated in glioma development, yet their prognostic relevance remains unclear. This study aimed to construct a robust prognostic model based on RNA processing factor genes and explore their functional roles and therapeutic potential. Transcriptomic and clinical data from glioma patients in the TCGA, CGGA, GEO and Rembrandt cohorts were analysed. Univariate, multivariate and LASSO‐Cox regression analyses were performed to establish a prognostic signature. Model performance was assessed using Kaplan–Meier survival curves, time‐dependent ROC analysis and C‐index evaluation. Key genes were identified via random forest analysis and validated through single‐cell datasets and immunohistochemistry. Functional assays were conducted to examine the biological roles of the key gene. Seventy‐eight RNA processing factor genes were associated with glioma prognosis, and a 19‐gene risk signature was constructed. The model effectively stratified patients into high‐ and low‐risk groups with significantly different survival outcomes (log‐rank *p* < 0.001). The AUCs for 1‐, 3‐ and 5‐year survival were 0.812, 0.774 and 0.769 in TCGA and 0.796, 0.758 and 0.741 in CGGA. The model achieved a C‐index of 0.781 and was validated as an independent prognostic factor. NSUN6 was identified as a key protective gene whose overexpression inhibited glioma cell proliferation and migration in vitro. RNA processing factor genes have prognostic utility in glioma. The 19‐gene model and NSUN6 highlight novel avenues for molecular stratification and targeted therapy.

**Abbreviations**
AUC: Area under curve
CGGA: Chinese Glioma Genome Atlas
CI: Confidence interval
CNS: Central nervous system
DCA: Decision curve analysis
GDSC: Genomics of dDrug sSensitivity in cCancer
GEO: Gene Expression Omnibus
GSEA: Gene set enrichment analysis
HPA: Human Protein Atlas
HR: Hazard ratio
HRD: Homologous recombination deficiency
IC50: Half‐ maximal inhibitory concentration
IDH: Isocitrate dehydrogenase
LOH: Loss of heterozygosity
MATH: Mutant‐allele tumor heterogeneity
mRNA: Messenger RNA
MSI: Microsatellite instability
*NSUN6*
: NOP2/Sun RNA methyltransferase 6
PRPFs: Prognostic RNA processing factors
ROC: Receiver operating characteristic
TCGA: The Cancer Genome Atlas
TIP: Tracking tumor immunophenotype
TISCH: Tumor immune single‐cell hHub
TMB: Tumor mutational burden
tRNA: Transfer RNA
WHO: World Health Organization

## 1 Introduction

Gliomas, among the most common malignant brain tumors, originate from glial cells within the central nervous system (CNS), and their prognosis is significantly correlated with tumor classification [1, 2]. The fifth edition of the World Health Organization (WHO) CNS tumor classification standards emphasizes molecular diagnostics, building upon the previous edition to enhance prognostic accuracy and therapeutic decision‐making for clinicians [3]. Gliomas are stratified into low‐grade (CNS WHO Grades I and II) and high‐grade (CNS WHO Grades III and IV), with glioblastoma representing the most aggressive phenotype and accounting for the majority of glioma‐related mortality [2, 4]. Despite treatments involving maximal safe surgical resection and adjunctive therapies such as radiotherapy, chemotherapy and electric field treatment, glioblastoma continues to exhibit a dismal 5‐year survival rate of less than 5% [5]. Although advances in epigenetics and transcriptomics have led to the identification of novel molecular markers, the need for effective prognostic tools and therapeutic targets remains urgent [1].

Molecular biomarkers such as *IDH1* mutation, *CDKN2A* homozygous deletion and 1p/19q codeletion are well‐established determinants of glioma classification and patient prognosis and have been incorporated into the WHO CNS5 guidelines [3]. While these features guide current clinical decision‐making, transcriptome‐derived prognostic models may offer complementary risk stratification and help uncover additional regulatory mechanisms beyond current histo‐molecular classification.

RNA processing factors are integral to post‐transcriptional gene regulation, participating in key steps such as RNA splicing, modification, polyadenylation and nuclear export [6, 7, 8]. These processes govern RNA stability and translation efficiency, thereby shaping cellular phenotypes. Aberrations in RNA processing have been increasingly linked to tumorigenesis, including dysregulated splicing, altered RNA methylation and defects in transcript surveillance [9, 10]. In gliomas, recent studies have identified abnormal expression of RNA methylation regulators and splicing factors, such as m6A‐associated proteins and serine/arginine‐rich splicing factors, as contributors to tumor progression and therapeutic resistance [11, 12, 13, 14, 15]. However, a comprehensive understanding of how RNA processing factor gene expression patterns affect glioma prognosis remains limited.

In this study, we systematically evaluated the expression and prognostic relevance of RNA processing factor genes across multiple glioma cohorts. We constructed and validated a robust 19‐gene prognostic model using five independent datasets, integrating survival data with transcriptomic and clinical variables. Functional enrichment analysis, immune landscape profiling and therapeutic correlation further elucidated the biological and clinical implications of the model. Among these genes, *NSUN6* emerged as a key protective factor, supported by single‐cell transcriptomics, protein expression data from the Human Protein Atlas and functional assays in glioma cell lines. These findings provide novel insights into the post‐transcriptional regulatory landscape of glioma and highlight RNA processing factors – particularly *NSUN6* – as promising prognostic markers and potential therapeutic targets.

Given their essential roles in RNA metabolism and emerging significance in glioma biology, RNA processing factor genes represent a promising yet underexplored class of prognostic biomarkers. Our study aimed to systematically characterise these genes and develop a predictive model that complements existing molecular classifiers and refines risk stratification for glioma patients.

## 2 Methods

### 2.1 Glioma Data Acquisition and Processing

First, we obtained gene expression profiles and corresponding clinical data of glioma patients from several repositories: The Cancer Genome Atlas (TCGA) (https://portal.gdc.cancer.gov/), the Chinese Glioma Genome Atlas (CGGA) (www.cgga.org.cn/), the Gene Expression Omnibus (GEO) (http://www.ncbi.nlm.nih.gov/geo/) and the Rembrandt database(www.cgga.org.cn/). We excluded patients with incomplete survival data from our analysis. To mitigate nonbiological variances and batch effects, we employed the ‘ComBat’ algorithm from the ‘sva’ package in *R*. ComBat uses an empirical Bayes framework to estimate and adjust for systematic biases across batches, making it particularly suitable for multi‐dataset integration where sample sizes and sequencing platforms vary. This method allows for effective removal of batch‐specific technical variation while preserving underlying biological heterogeneity, thus ensuring the comparability of transcriptomic data derived from TCGA, CGGA, GEO and Rembrandt cohorts. RNA processing factor genes were obtained from the AmiGO database(http://amigo.geneontology.org/amigo).

### 2.2 Construction, Evaluation and Application of the RNA Processing‐Related Signature

We utilized the ‘survival’ package to conduct univariate Cox and Kaplan–Meier (K‐M) survival analyses on the TCGA‐glioma, CGGA‐693, CGGA‐325 and GSE16011 datasets. We applied a stringent *p* value threshold of less than 0.001 to ensure the reliability of the results. Subsequently, multivariate Cox analyses were conducted with a *p* value threshold of less than 0.05, identifying prognostic RNA processing factor genes as characteristic markers. Using the TCGA dataset as a training cohort, we employed LASSO‐Cox regression to refine our model by eliminating genes with collinear expression patterns.

The risk score model was established by combining the expression levels of PRPFs, each weighted by its respective coefficient (*β*), calculated as follows: Risk Score = (β1 * PRPF1 + β2 * PRPF2 + β3 * PRPF3 + … + βn * PRPFn). Patients in the training set were stratified into high‐risk and low‐risk groups based on the median risk score, and their survival outcomes were compared using Kaplan–Meier (K–M) analysis and the log‐rank test. The model's discriminative ability and accuracy were evaluated using receiver operating characteristic (ROC) curve analysis and calibration curve analysis. For validation, datasets from the CGGA and GEO were used inernally, with the Rembrandt dataset serving as an external validator to ascertain the model's efficacy in predicting survival outcomes.

Further analyses explored the prognostic significance of clinical variables – including age, gender and pathology grade – as well as the risk score, through both univariate and multivariate Cox regression analyses within the training cohort. These variables were selected based on their consistent availability across public datasets. However, other important clinical indicators such as the extent of surgical resection and preoperative Karnofsky Performance Status (KPS) were excluded from this study due to their inconsistently or missing reporting across the TCGA, CGGA, GEO and Rembrandt cohorts. Model prediction accuracy was quantified using time‐dependent C‐index curves. Decision curve analysis (DCA) was also performed to evaluate the clinical utility of the prognostic model.

### 2.3 Exploring Potential Mechanisms

In this study, glioma patients were divided into high‐risk and low‐risk groups based on median risk scores. We initiated our analysis by highlighting significant heterogeneities between these groups through the examination of various biological mechanisms and signalling pathways. Using radar charts, we illustrated the relationships between the risk scores and genomic heterogeneities in glioma patients, including aspects such as tumor mutational burden (TMB), tumor purity and chromosomal instability. Additionally, an exploratory analysis of methylation‐related genes in the TCGA dataset was conducted, with bar graphs depicting the correlation between risk scores and gene methylation status, including DNA, histone and m6A methylation (*p* < 0.05). Subsequently, heatmaps created with the ‘Complex Heatmap’ package in R were used to display the associations between risk scores and Tracking Tumor Immunophenotype (TIP) scores. To further explore the biological disparities between the high‐risk and low‐risk groups, GSEA was used to identify tumor characteristics linked with the risk scores. Finally, a suite of immunological profiling tools such as TIMER, xCELL, quanTIseq, MCPcounter, CIBERSORT, CIBERSORT‐ABS and EPIC was used to measure the immune infiltration in these patients [16, 17, 18, 19, 20, 21, 22]. This study revealed notable differences in immune infiltration across the groups and confirmed correlations between the risk score and immune cell content.

### 2.4 Predicting Chemotherapy Drug Response

As chemotherapy is a commonly used treatment for glioma, we used the ‘pRRophetic’ *R* package [23], leveraging the Genomics of Drug Sensitivity in Cancer (GDSC) database, to calculate and compare the half‐maximal inhibitory concentrations (IC50) of chemotherapy drugs between high‐risk and low‐risk groups. Additionally, we assessed the correlation between the IC50 values and the risk scores, providing insights into chemotherapeutic responsiveness relative to patient risk stratification.

### 2.5 Identification of the Key Genes

We ranked the genes in the model based on survival importance by a random forest algorithm.

The gene with the highest importance was considered to be the key gene, and a meta‐analysis across various datasets further elucidated the implications of these key genes for survival. Additionally, we analysed the expression of key genes at the single‐cell level using the Tumor Immune Single‐Cell Hub (TISCH) database. GSEA highlighted the differential activation of signalling pathways associated with key genes between the risk groups. Using the Human Protein Atlas (HPA), we further detailed the protein‐level expression disparities of these key genes, enhancing our understanding of their biological impact.

### 2.6 Cell Lines and Reagent Preparation

The human glioma cell lines, LN229 and U251, were obtained from the Cell Bank of the Chinese Academy of Sciences (Shanghai, China). The cells were cultivated in a complete culture medium composed of 90% Dulbecco's modified Eagle's medium (DMEM), 10% fetal bovine serum (FBS) and 1% penicillin–streptomycin solution.

### 2.7 Gene Overexpression

The RNA overexpression plasmid was constructed by Nanjing Corues Biotechnology Co. Ltd. LN229 and U251 cells were separately seeded in 6‐well plates, and upon reaching approximately 80% confluence, they were transfected with the RNA overexpression plasmid using Lipofectamine 3000 reagent (Thermo Fisher Scientific) according to the manufacturer's protocol. To assess the overexpression efficiency, transfected cells were collected for subsequent qRT–PCR and western blotting at 48 h after transfection.

### 2.8 Quantitative Real‐Time PCR

Total RNA was extracted using Trizol reagent (Invitrogen, USA) based on the manufacturer's protocol. Quantification of RNA concentration was performed using NanoDrop One. 1.5 μg RNA was transferred to cDNA using Evo M‐MLV RT Kit (AG, China). The SYBR Green PCR Mastermix Kit (AG, China) was used to perform PCR amplification on QuantStudio Real‐Time.

PCR software. 2‐DDCt value was calculated to perform the quantitative analysis. The primers are as follows:

*NSUN6* forward: 5′‐TTAAGAGGAGCCCATGTCTATGC‐3′,

*NSUN6* reverse: 5′‐CTTTGCGGCTTAGTTCAGAAATC‐3′;

GAPDH was used as an internal control.

### 2.9 Western Blot

Cells were harvested and lysed with RIPA lysis buffer (P0013B, Beyotime). The concentrations of the proteins were quantified by a BCA protein quantification kit (P0010, Beyotime). Equivalent amounts of protein samples (10 μg per lane) were electrophoretically separated by 10% SDS‐PAGE and then transferred to PVDF membranes (IPVH00010, Millipore, Massachusetts, USA). The membranes were blocked for 10 min at room temperature with NcmBlot blocking buffer (P30500, NCM Biotech, Suzhou, China), followed by washing membranes with Tris‐buffered saline containing 0.1% Tween‐20 (TBST). Then, the membranes were incubated at 4°C overnight with specific primary antibodies (anti‐*NSUN6*, 17,240‐1‐AP, Proteintech, 1/1000; anti‐GAPDH, 60004‐1‐Ig, Proteintech, 1/10000). The next day, a species‐matched secondary antibody (Nakasugi Jinqiao, 1/10000)was used to incubate the membrane for 1 h at room temperature. Finally, wash again with TBST shock for 10 min and repeat three times. It was blotted dry with absorbent paper and then moistened with the developing solution and placed in the imager for exposure.

### 2.10 Cell Counting Kit‐8 (CCK‐8)

48 h post‐transfection, LN229 and U251 cells were harvested using trypsin digestion. After digestion was terminated, the cells were centrifuged, the supernatant was discarded and the cells were resuspended in 1 mL of complete medium to prepare the cell suspension. Using a 20 μL pipette tip, 20 μL of the cell suspension was taken and added to both ends of a haemocytometer for counting. The appropriate dilution was calculated to achieve a density of approximately 5000 cells per 100 μL. The cell suspension (100 μL) was seeded in each well of a 96‐well plate, with three replicate wells per condition. Around 100 μL of PBS was added to the surrounding wells to minimise evaporation. Cell viability was assessed at 0 h, 24 h, 48 h and 72 h after cell adherence. The medium was discarded, and PBS from five wells was also discarded to serve as blank controls. Each well then received 100 μL of complete medium mixed with 10 μL of CCK‐8 solution, and the plates were incubated in the dark in the cell culture incubator. Measurement: Absorbance at 450 nm (OD values) was measured at 0.5 h, 1 h, 2 h and 4 h after adding the CCK‐8 solution.

### 2.11 Colony Formation Assay

Five hundred cells were inoculated in a six‐well plate, followed by timely replacement of the medium according to the cell morphology and growth condition. The above six‐well plate was cultivated for 10–14 days until the cell mass appeared at the bottom of the plate. After washing with PBS twice, 4% paraformaldehyde was added to fix for 15 min. 1% Crystal violet was subsequently added to the plate to stain for a further 30 min while the number of colony formations was counted and recorded. The experiment was repeated three times.

### 2.12 Scratch Assay

Cells at the logarithmic growth stage were inoculated in a six‐well plate. When the cell density reached 80%–90%, the cells were scratched perpendicularly at a constant speed with a 1 mL gun head, cleaned twice with PBS and photographed under a microscope, and the scratch area of 0 h was recorded. After the serum‐free medium was added for another 6 h, 12 h and 24 h, the scratch area was photographed again to record. The cell mobility was calculated by comparing the change of scratch area. The experiment was repeated three times.

### 2.13 Statistical Analyses

Statistical analyses were conducted using *R* software (version 4.3.0) and SPSS (version 25.0; IBM SPSS Inc., Armonk, NY). During data preprocessing, duplicate values were averaged, missing values were removed and RNA‐seq data were merged and normalised using the ‘sva’ package in R. Kaplan–Meier survival analysis and log‐rank tests were employed to evaluate patient outcomes and prognostic comparisons among different groups. Spearman's method was chosen for correlation analysis due to its robustness in non‐parametric data assessment; clinical variables were compared using Wilcoxon tests for non‐parametric data and t‐tests for parametric data. Cox regression analysis, conducted using the ‘survival’ *R* package, was used to evaluate survival outcomes; the model's diagnostic ability was assessed using ROC curves from the ‘survival ROC’ package. To reduce false positives in high‐dimensional univariate screening, a stringent threshold of *p* < 0.001 was applied in the initial Cox analysis. For subsequent multivariate Cox regression, a conventional *p* < 0.05 threshold was adopted to evaluate the independent prognostic contribution of selected genes. Relative risks were quantified by hazard ratios (HR) with 95% confidence intervals (CI). Cellular experiment data were expressed as mean ± standard deviation (SD) and tested for distribution normality using the Kolmogorov–Smirnov test. One‐way ANOVA was used to discern statistical differences across multiple groups, with a significance threshold set at *p‐value* < 0.05.

## 3 Results

Based on increasing evidence linking aberrant RNA processing to glioma progression, immune evasion and therapy resistance, we hypothesized that RNA processing factor genes may contain prognostically informative signals. To test this, we first curated a set of genes involved in RNA splicing, methylation, stability and translation regulation and then evaluated their association with overall survival (OS) using a multi‐step statistical pipeline.

### 3.1 Data Retrieval and Preprocessing

Our study began with the download of gene expression profiles and associated clinical information for glioma patients from the TCGA database. Glioma patients with comprehensive follow‐up data and who were tracked for more than 30 days post‐diagnosis were included, to ensure robust longitudinal analysis. The same selection criteria were applied to extract data from the CGGA‐693, the CGGA‐325, the GSE16011 and the Rembrandt datasets. To address potential batch effects due to non‐biological variations among these datasets, the ‘ComBat’ algorithm from the *R* package ‘sva’ was utilized. This step was crucial for harmonizing data across studies and minimizing variability unrelated to biological differences. Additionally, our analytical framework was enriched by incorporating a gene set from the AmiGO database, which included 929 genes identified as human RNA processing factors.

### 3.2 The 19‐Gene Signature is an Independent Prognostic Factor for Glioma

We first performed univariate Cox and Kaplan–Meier (K‐M) survival analyses on four glioma cohorts (TCGA, CGGA‐693, CGGA‐325 and GSE16011) using the ‘survival’ package in *R*, applying a stringent threshold of *p* < 0.001 to enhance the reliability of prognostic gene selection. This yielded 478 candidate genes associated with overall survival. Subsequently, multivariate Cox regression with *p* < 0.05 was used to narrow the selection to 78 prognosis‐related RNA processing factor genes.

**FIGURE 1:**
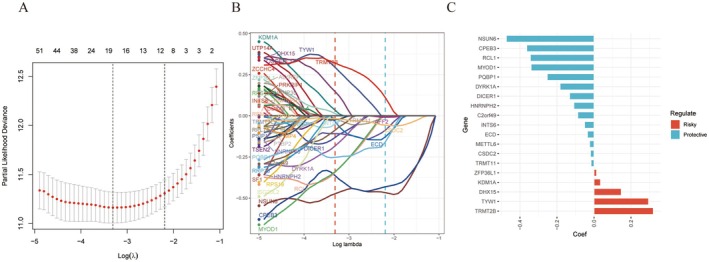
Construction of the RNA processing factor gene‐related risk signature model. (A, B) Partial likelihood deviance of variables revealed by the LASSO regression model. The red dots represent the partial likelihood of deviance values, the grey lines represent the standard error (SE); and the two vertical dotted lines on the left and right represent the optimal values according to the minimum criteria and 1‐SE criteria, respectively. (C) Nineteen RNA processing factor genes used to construct the prognostic model and their coefficients.

Using the TCGA cohort as the training set, we applied LASSO‐Cox regression to reduce the model complexity and eliminate multicollinearity among variables (Figure 1A–C). As shown in Figure 1A, cross‐validation identified the optimal value of the regularization parameter (*λ*). Figure 1B illustrates the LASSO coefficient paths of the 78 candidate genes across different *λ* values. Nineteen genes were ultimately selected (Figure 1C), including protective and risk‐associated factors, to construct a prognostic signature. The full gene list and coefficients are available in Table S1.

**FIGURE 2:**
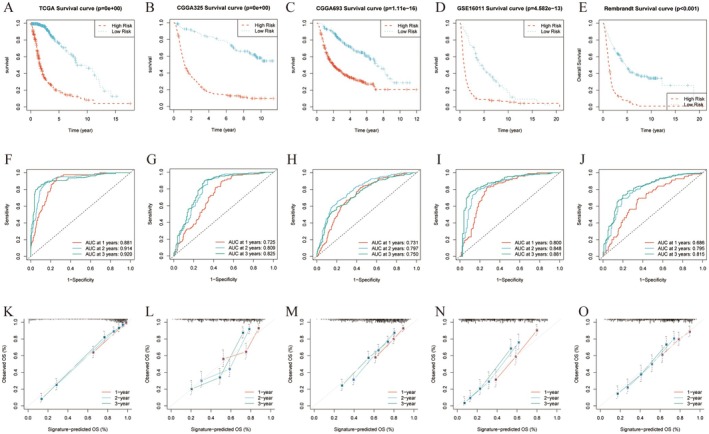
Survival analysis, ROC analysis and DCA analysis of two risk groups associated with the 19‐gene signature in the TCGA training cohort (A, F, K), three internal validation cohorts (B‐D, G‐I, L‐N) and one external validation cohort (E, J, O).

We then divided the glioma patients into a high‐risk group and a low‐risk group using the median risk score as the threshold. The K‐M curves revealed that the overall survival was significantly better in the low‐risk group than in the high‐risk group (Figure 2A–E). The AUCs for 1‐, 2‐ and 3‐year OS predicted by the model had satisfactory specificity and sensitivity, validating the survival prediction performance of the prognostic model (Figure 2F–J). The calibration curves demonstrated agreement between model‐predicted survival and the actual observed survival (Figure 2K–O).

**FIGURE 3:**
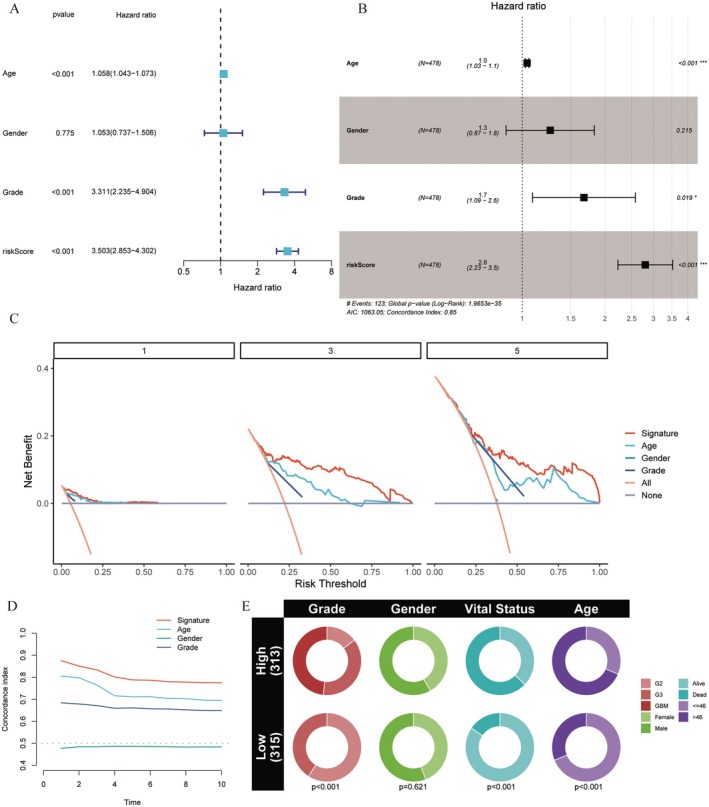
Independence assessment, decision curve analysis and calibration of the constructed risk prediction model. (A, B) Independent prognosis‐related factors identified through Cox regression analysis. (C) The y‐axis measures the net benefit. The red line represents the constructed risk prediction model; the grey line indicates that no indicators were used to predict all patients; and the orange line indicates that all indicators were used for patient predictions. The decision curve demonstrated that the constructed risk prediction model provided greater benefit in predicting glioma patient survival than either the indicator‐all‐patients scheme or the indicator‐none scheme. (D) C‐index estimates the probability that the predicted results are consistent with the actual observed results. (E) Pie chart illustrates differences in glioma stage, gender, survival status and age between the high‐ and low‐risk groups.

Univariate Cox regression and multivariate Cox analysis suggested that the prognostic models were independent prognostic factors for glioma patients (Figure 3A,B) (*p* < 0.001), and the time‐dependent C‐index indicated that the prognostic model had superior predictive accuracy compared to traditional clinical variables for the prognosis for glioma patients, implying that our signature was very robust (Figure 3C). The DCA analysis suggested that the use of prognostic models to predict patient survival may benefit patients more than traditional clinical variables (Figure 3D,E).

### 3.3 Exploring Potential Mechanisms

**FIGURE 4:**
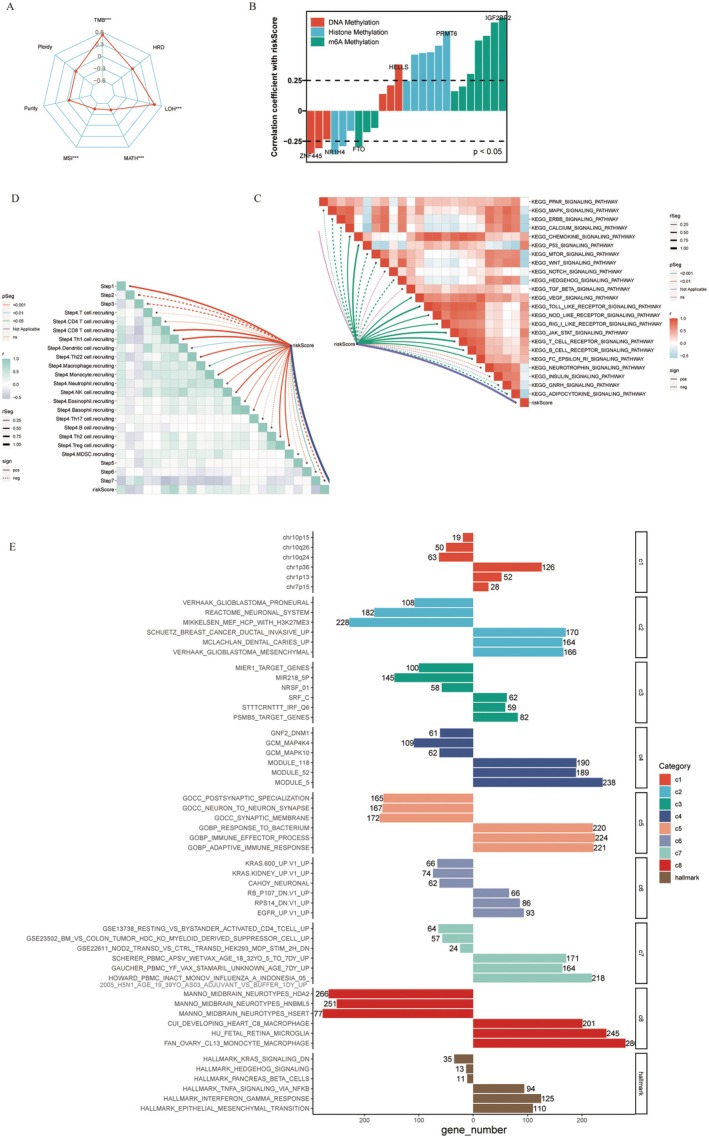
Correlation analysis used to identify potential mechanisms. (A) The radar chart illustrates the correlation between the risk scores and genomic heterogeneity in glioma patients. (B) The bar graph depicts the correlation coefficients between the risk scores and DNA, histone and m6A methylation (*p* < 0.05). (C) The butterfly chart demonstrates the correlation between the risk scores and each phase of the TIP score. (D) The butterfly chart reveals the correlation between the risk scores and various signaling pathways. (E) The bar graph illustrates the primary gene enrichment trends identified through GSEA.

Our prognostic model effectively quantifies glioma patient risk, offering substantial clinical value. To elucidate the significant heterogeneity between the high‐risk and low‐risk groups, we conducted a correlation analysis to identify potential mechanisms influencing glioma prognosis. Utilising radar charts, we correlated the risk scores with several indicators of genomic heterogeneity, including TMB and tumor purity. The analysis revealed that the risk score was strongly positively correlated with TMB and LOH, and negatively with MATH and MSI (Figure 4A). Further correlation analysis of methylation‐related genes indicated strong positive correlations between the risk score and HELLS (DNA methylation‐related) and IGF2BP2 (m6A methylation‐related), highlighting potential targets for further research (Figure 4B). Additionally, we utilized a butterfly chart to vividly depict the interactions between the risk score and the TIP score across various stages of the immune response, from antigen release to cancer cell destruction. Notably, the risk score was positively correlated with the immune cell recruitment during the early and intermediate stages of the immune response (Steps 1 and 4, Figure 4C). This analysis provides insights into how the risk score might influence immune dynamics and patient outcomes in glioma patients, supporting the use of our model in clinical settings to predict and improve glioma patient prognosis.

In our study, we employed GSEA to discern the tumor biological characteristics correlated with the prognostic risk score (Figure 4D). This analysis revealed a significant positive correlation between the risk score and several key signaling pathways. Notably, the VEGF signaling pathway, known for its role in promoting angiogenesis and tumor growth, and the Toll‐like receptor signaling pathway, integral to mediating immune responses, were both positively associated with higher risk scores.

To investigate whether glioma patients in the high‐risk and low‐risk groups are biologically heterogeneous, we also performed GSEA to identify tumor characteristics associated with risk scores (Figure 4E). GSEA demonstrated that the risk score is significantly associated with the immune response, neurological response, KRAS signaling pathway and glioblastoma‐related tissues.

**FIGURE 5:**
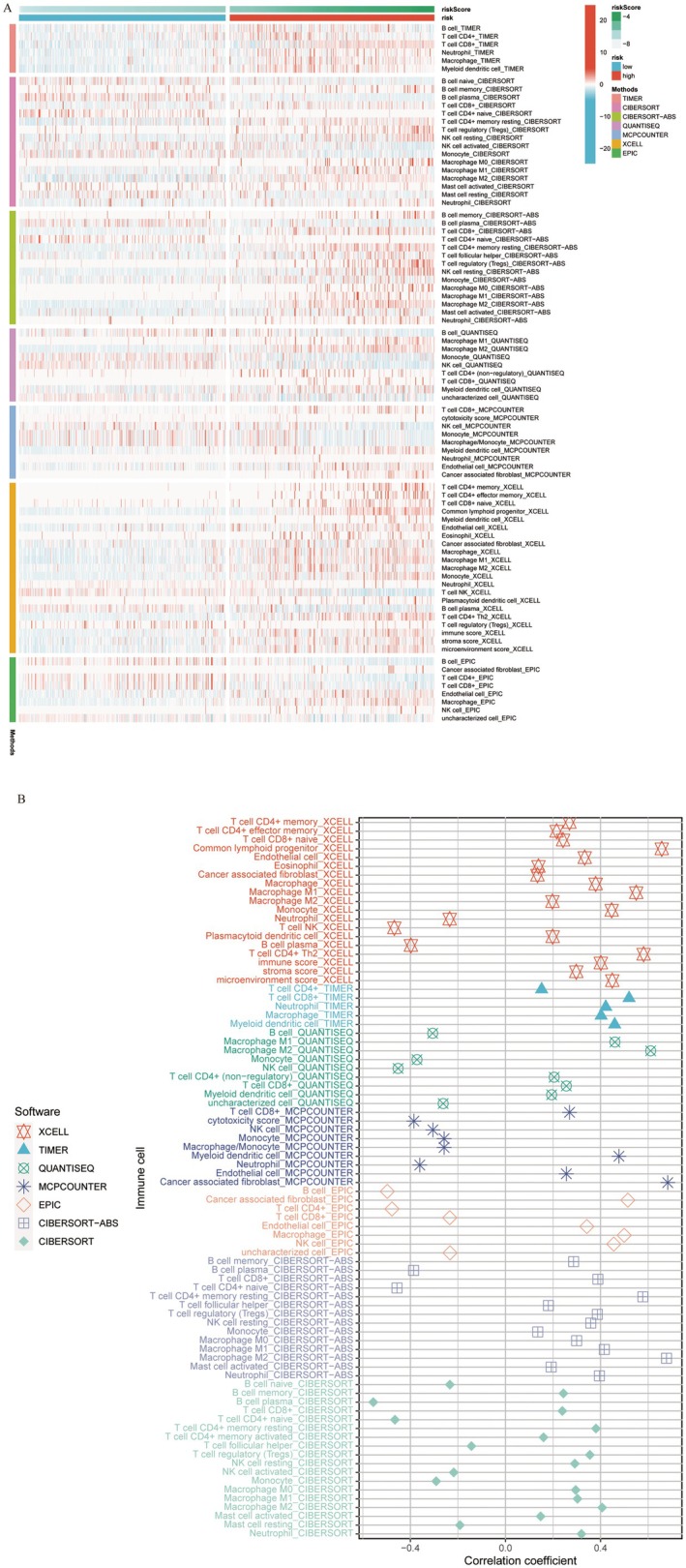
Correlation between the risk score and immune infiltration. (A) Differential heatmap illustrating the relationship between the risk score and immune infiltration across various algorithms. (B) Correlation plot illustrating the association between risk score and various types of immune cells.

To further understand the relationship between the risk score and immune pathways, we quantified the abundance of patient immune infiltration using algorithms such as TIMER, xCELL, quanTIseq, MCPcounter, CIBERSORT, CIBERSORT‐ABS and EPIC and compared the differences in immune infiltration between the high‐risk and low‐risk groups. According to these immune infiltration algorithms, patients in the high‐risk group exhibited greater immune cell infiltration (Figure 5A). Additionally, the risk score was significantly positively correlated with several tumor‐immune‐related cell types, including M1 macrophages, M2 macrophages, CD4+ T cells, neutrophils and myeloid dendritic cells (Figure 5B).

### 3.4 The 19‐Gene Signature Has Significant Application Value

**FIGURE 6:**
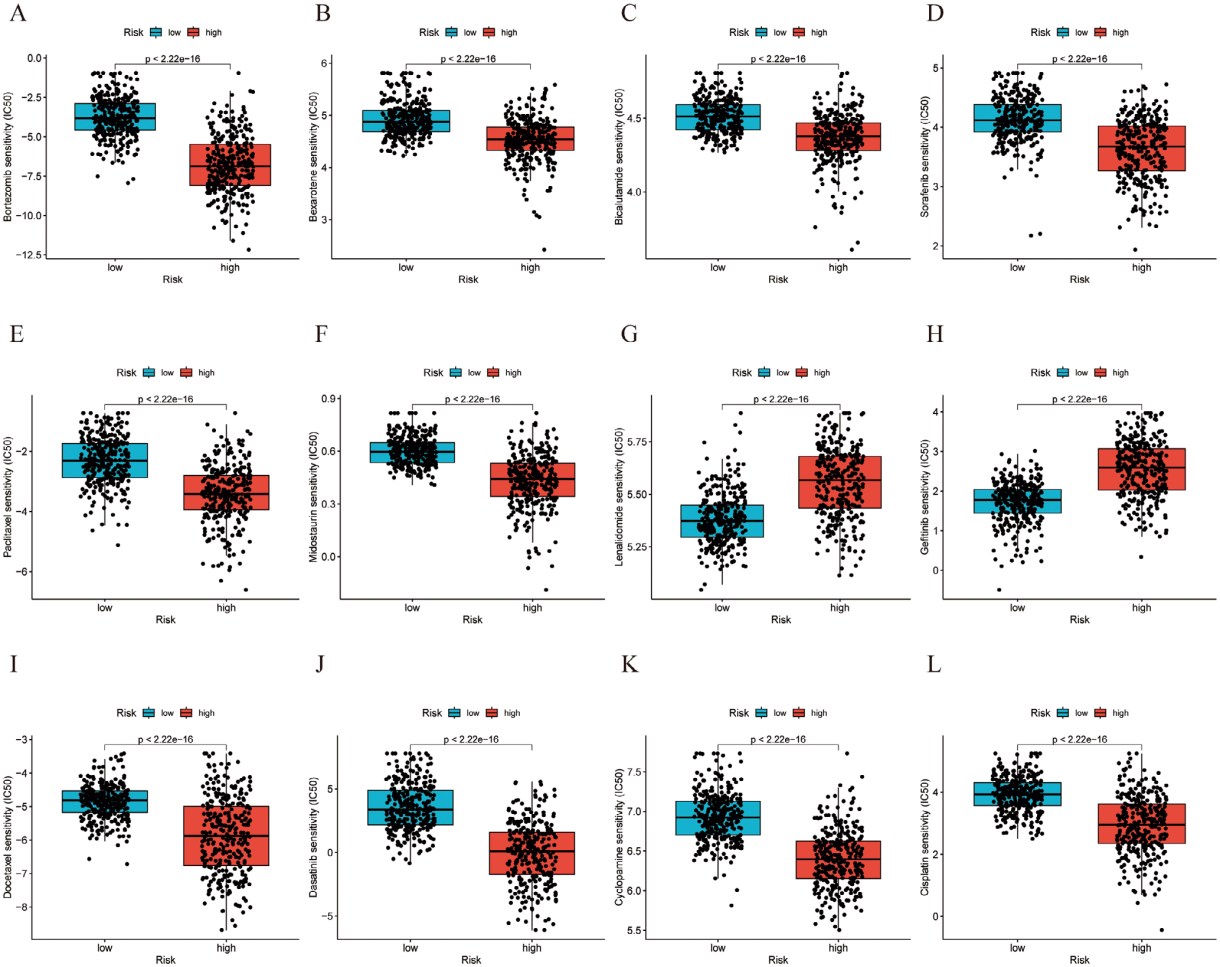
Chemotherapeutic responses in high‐ and low‐risk glioma patients. (A) Bortezomib; (B) Bexarotene; (C) Bicalutamide; (D) Sorafenib; (E) Paclitaxel; (F) Midostaurin; (G) Lenalidomide; (H) Gefitinib; (I) Docetaxel; (J) Dasatinib; (K) Cyclopamine; (L) Cisplatin.

Utilizing the GDSC database, we calculated and compared the IC50 values of chemotherapy drugs between high‐risk and low‐risk glioma patients and analysed their correlation with risk score to guide clinical drug selection. Lower IC50 values indicate greater sensitivity to drugs. The prognostic model's drug sensitivity analysis identified 55 drugs with significant differences in sensitivity; 12 common uesd drugs were selected for display and discussion (Figure 6). The risk score was negatively correlated with sensitivity to lenalidomide and gefitinib, and positively correlated with bortezomib, cisplatin, pemetrexed, dasatinib, docetaxel, mitotane, paclitaxel and sorafenib, suggesting that patients with higher risk scores respond better to these drugs. This finding offers valuable insights for the personalized treatment of glioma patients.

### 3.5 Identification and Analysis of the Key Gene

**FIGURE 7:**
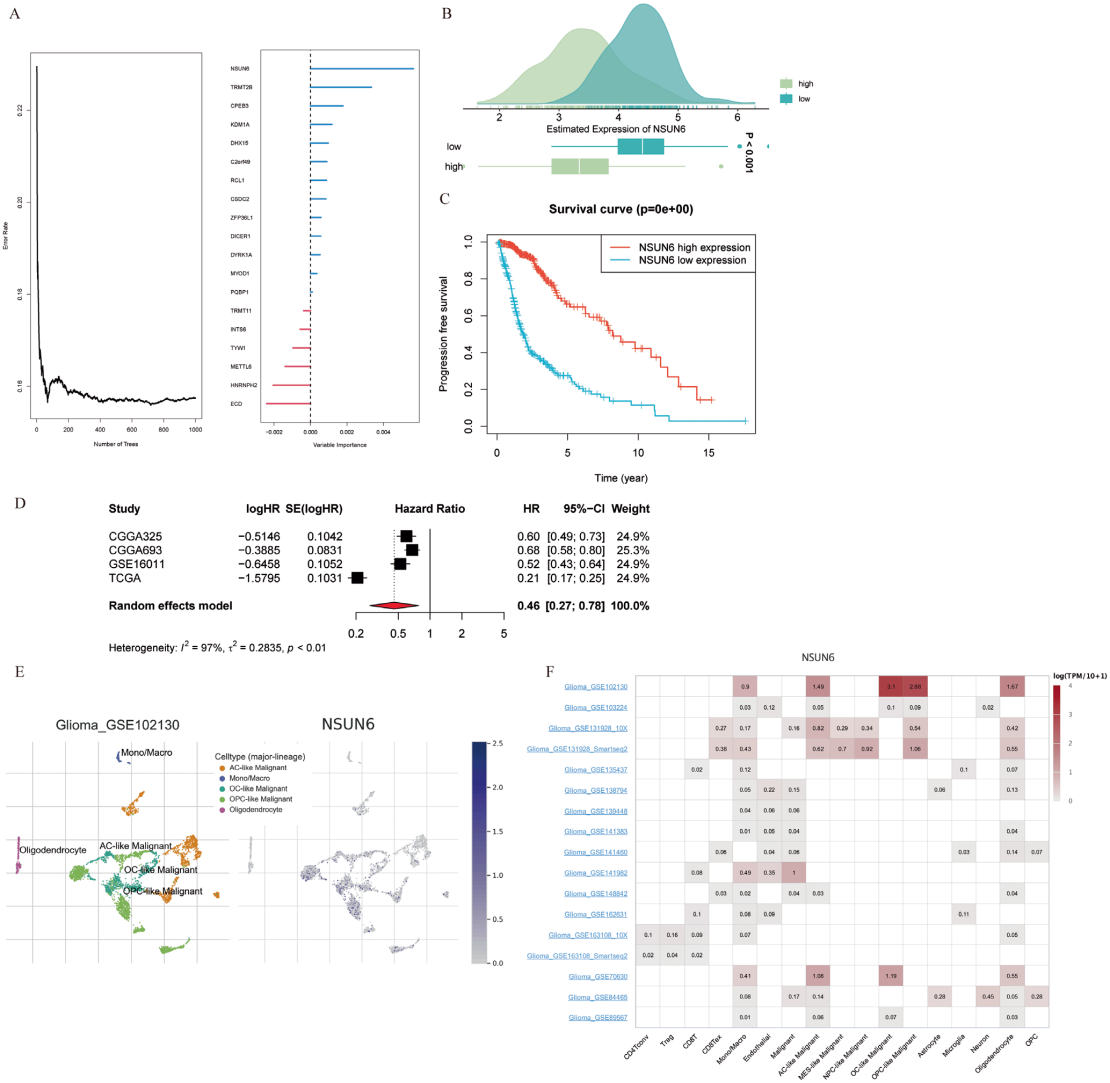
Identification, survival analysis and localization of the *NSUN6* at the single‐cell level. (A) The survival significance of 19 genes in the model was ranked, and key genes were identified using the random forest algorithm. (B) The ridge plot suggests that *NSUN6* is differentially expressed between the high‐ and low‐risk groups (*p* < 0.001). (C) Survival curves for the high‐ and low‐*NSUN6*expression groups show a significant difference (*p* < 0.001). (D) Meta‐analysis confirmed the consistent effect of *NSUN6* on survival across five datasets. (E) Expression of the *NSUN6* in various malignant cell subtypes was analyzed using the GSE102130 dataset. (F) Hotspot maps of *NSUN6* gene expression across various malignant cell subtypes were analysed in single‐cell data from multiple glioma patients.

We utilized the random forest algorithm to rank the genes in the model by survival importance, identifying the *NSUN6* as the most critical (Figure 7A). Subsequently, we observed significant differential expression of the *NSUN6* between the high‐risk and low‐risk groups, with higher expression in the low‐risk group (Figure 7B). We then stratified patients into high‐ and low‐*NSUN6* expression groups for survival analysis and found that patients with high *NSUN6* expression had significantly better OS than those with low *NSUN6* expression (Figure 7C). Further meta‐analysis confirmed that the *NSUN6* was associated with improved survival across five datasets, indicating a potential protective role (Figure 7D). To evaluate *NSUN6* expression across different cell lines, we analyzed its expression at the single‐cell level using the TISCH database. *NSUN6* was widely expressed among malignant cell types, particularly in astrocyte‐like, oligodendrocyte‐like and oligodendroglia precursor‐like cells (Figure 7E,F).

**FIGURE 8:**
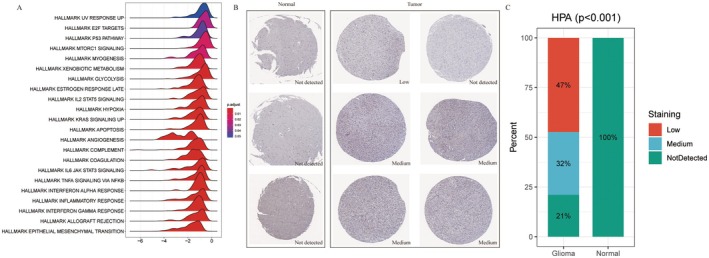
*NSUN6* gene enrichment and differential expression in normal and tumor samples. (A) Ridge plot displaying GSEA results for the *NSUN6* gene. (B) HPA immunohistochemical staining revealed moderate *NSUN6* staining in glioma tissues. (C) Histogram illustrating a significant difference in *NSUN6* staining intensity between normal and glioma tissues (*p* < 0.001).

Through GSEA, we further explored the activation status of *NSUN6*‐related signaling pathways in the high‐ and low‐risk groups, revealing significant differences in biological processes and signaling pathways, including the E2F‐targeting, P53 and mTORC1 signaling pathways; glycolysis; and xenobiotic metabolism (Figure 8A). We next analyzed the differential protein expression of *NSUN6* using the HPA database. Compared to normal tissues, various cancer samples, particularly glioma samples, exhibited significant differences in the expression of this gene (Figure 8B). Additionally, a chi‐square test of the HPA data revealed significant differences in *NSUN6* expression between glioma and normal tissues (Figure 8C).

### 3.6 NSUN6 Overexpression Inhibits the Progression of Glioma In Vivo

**FIGURE 9:**
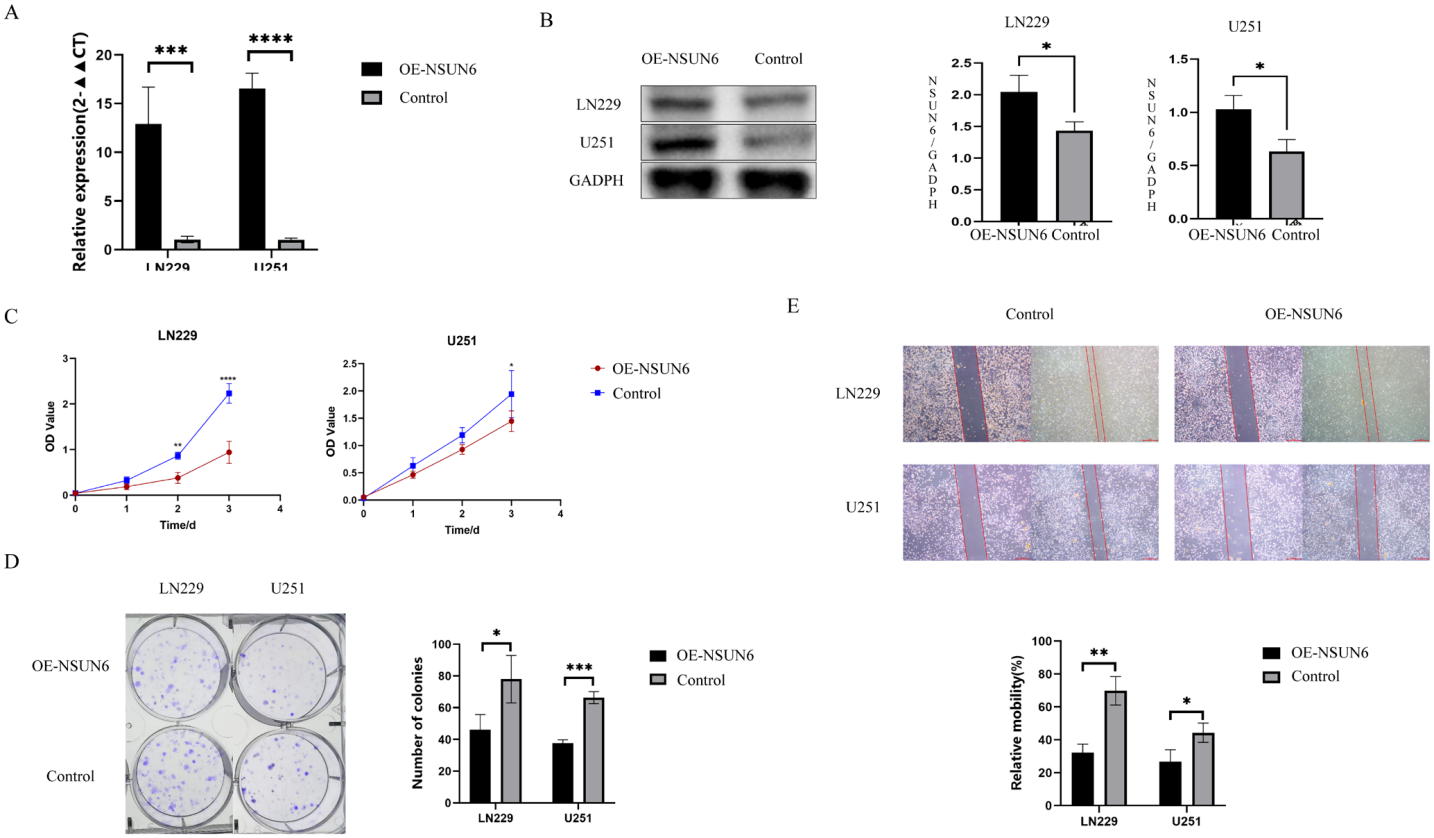
Effect of *NSUN6* overexpression on the proliferation and migration of LN229 and U251 glioma cell lines. (A) After *NSUN6* plasmid transfection, *NSUN6* mRNA levels were significantly elevated in LN229 and U251 glioma cells. (B) Following *NSUN6* plasmid transfection, the protein levels of *NSUN6* were significantly increased in LN229 and U251 glioma cells. (C) CCK‐8 assays showed that the proliferation of LN229 and U251 glioma cells was significantly inhibited following *NSUN6* overexpression. (D) Clonogenic assays confirmed that clonal growth of LN229 and U251 glioma cells was significantly inhibited following *NSUN6* overexpression. (E) Scratch assays indicated that the migration of LN229 and U251 glioma cells was significantly reduced following *NSUN6* overexpression. Each experimental group consisted of three replicates (*n* = 3), with statistical significance noted as *p < 0.05, **p < 0.01, ***p < 0.001 and ****p < 0.0001.

To verify the role of *NSUN6* expression in glioma cells, we constructed an *NSUN6* overexpression plasmid and conducted experiments on the LN229 and U251 glioma cell lines. Following *NSUN6* plasmid transfection, both mRNA and protein expression were significantly elevated, confirming overexpression at the transcriptional and translational levels (Figure 9A,B). After confirming successful transfection, we assessed cell proliferation and migration abilities of the *NSUN6*‐overexpression and negative control groups (transfected with control plasmid). The CCK‐8 assayshowed that the proliferation of the *NSUN6‐*overexpressing group was significantly lower than that of the negative control group (Figure 9C). To further validate the effects in cell proliferation, we performed clonogenic assays, which yielded similar results (Figure 9D). Subsequently, to evaluate the effects of *NSUN6* overexpression on cell migration, we performed scratch assays and revealed that migration in the *NSUN6* overexpression group was significantly inhibited compared to that in the negative control group (Figure 9E).

## 4 Discussion

In this study, we identified 78 RNA processing factor genes associated with glioma prognosis and constructed a 19‐gene prognostic signature using LASSO‐Cox regression. These genes were further categorized as protective or risk‐associated groups based on their hazard ratios. Notably, *NSUN6* emerged as the most influential protective factor, followed by other tumor‐suppressive genes such as *CPEB3*, *RCL1* and *DYRK1A*.

Protective genes, such as *NSUN6*, *CPEB3*, *DICER1* and *RCL1*, are broadly involved in RNA methylation, mRNA translation regulation and ribosomal RNA processing. *CPEB3* has been shown to inhibit the progression of gastrointestinal and hepatic cancers by regulating mRNA translation, whereas *RCL1* is suggested to act as a tumor suppressor via ribosome biogenesis modulation. *DYRK1A* and *DICER1* are involved in neural development and miRNA processing, both of which are dysregulated in glioma.

In contrast, risk‐associated genes such as *DHX15*, *KDM1A* and *ZFP36L1* are involved in RNA helicase activity, chromatin modification and inflammation‐related pathways. *DHX15*, though reported to act as a tumor suppressor in some cancers, may contribute to glioma progression via androgen receptor signaling and warrants further study.

To deepen our understanding of the functional relevance of the model components, we conducted a literature‐based survey of selected model genes. Below, we summarize additional evidence supporting their roles in tumor biology, focusing on their potential contributions to glioma pathogenesis and progression. *CPEB3* encodes an RNA‐binding protein that regulates mRNA translation through multiple mechanisms and has been implicated in gastrointestinal tumors [24]. It exerts a protective role by suppressing the progression of gastric and hepatocellular carcinomas through inhibition of mRNA translation [25, 26]. Notably, reduced *CPEB3 activity* in gliomas may contribute to tumor progression and malignancy [27]. *RCL1* encodes an endonuclease involved in processing pre‐rRNA [28] and has recently been proposed as a novel tumor suppressor in hepatocellular carcinoma [29]. Although its expression in glioma remains unexplored, its biological function suggests a potential tumor‐suppressive role in the CNS as well. Other protective genes, including *DYRK1A*, *DICER1*, *MYOD1* and *INTS6*, also contribute to tumor suppression through diverse mechanisms. *MYOD1* mutations are associated with poor prognosis in rhabdomyosarcoma [30]; *DYRK1A* is a conserved kinase implicated in the pathogenesis of several diseases, including cancer and neurodegeneration [31]; *DICER1*, a key RNA‐processing endonuclease, is linked with hereditary tumor susceptibility syndromes [32] and *INTS6* regulates gene expression through premature transcriptional termination and functions a context‐dependent tumor regulator [33, 34].

Additionally, *METTL6* and *TRMT11* encode tRNA methyltransferases that regulate transcript processing and promote tumor cell proliferation [35, 36]. Although *HNRNPH2* is primarily linked to neurodevelopmental disorders, may be functionally relevant in glioma, though its oncogenic potential remains unclear [37]. The functions of *PQBP1*, *C2orf49*, *ECD* and *CSDC2* in tumorigenesis remain largely uncharacterised and warrant further investigation.

Among the risk‐associated genes, *DHX15* was identified as having a particularly strong impact on the risk score. It encodes an RNA helicase involved in transcript splicing and RNA surveillance. Although previously reported as a tumor suppressor in glioblastoma [38], *DHX15* has also been shown to promote prostate cancer by enhancing androgen receptor transcriptional activity [39], and its depletion has been linked to leukemia pathogenesis [40]. These findings suggest that *DHX15* may function as a context‐dependent regulator in glioma, and its precise role warrants further investigation.

Other risk genes include *KDM1A*, a histone demethylase implicated in oncogenic chromatin remodeling [41], and *ZFP36L1*, an RNA‐binding protein associated with inflammation and gastric cancer development [42]. While the roles of *TRMT2B* and *TYW1* in cancer are not yet defined, their significant coefficients in our model suggest potential functional importance that warrants further study.

Collectively, the inclusion of these 19 genes – many functionally involved in RNA metabolism, chromatin modification and translational regulation – supports their relevance to glioma prognosis. Further mechanistic investigations may provide a foundation for therapeutic development targeting these RNA processing factors.

After establishing the prognostic model, we evaluated its predictive accuracy and robustness across multiple datasets, including the training set, internal validation set and external validation set, using K‐M survival analysis, ROC curve analysis and calibration curve analysis. K‐M survival analysis revealed that patients in the high‐risk group consistently had significantly shorter overall survival compared to those in the low‐risk group according to the TCGA, CGGA, GEO and Rembrandt datasets, demonstrating the high stability of the model. ROC and calibration curve analyses indicated that the prognostic model effectively predicted glioma patient survival at 1, 2 and 3 years. Both univariate and multivariate Cox analyses confirmed that the prognostic model is an independent predictor of overall survival in gliomas. C‐index curves demonstrated that the prognostic model outperformed traditional clinical variables in survival prediction. DCA indicated that using the prognostic model to predict patient survival potentially offers greater clinical value than traditional clinical variables. These findings suggest that the prognostic model, based on RNA processing factor genes, provides superior predictive capabilities, offering more accurate survival predictions for glioma patients and potentially benefiting clinical decision‐making.

Subsequently, we analysed the genomic heterogeneity between the high‐ and low‐risk groups, differences in tumor pathways and variations in methylation‐related genes, providing fresh insights into the biological behavior of gliomas. Furthermore, we explored the connection between the prognostic model and the tumour immune microenvironment and identified chemotherapeutic agents potentially sensitive to different patient groups through drug sensitivity analysis in the high‐ and low‐risk groups. Lenalidomide, an immunomodulator effective against haematopoietic cell tumours, has shown preliminary antitumour effects in gliomas through cellular experiments [43]. Gefitinib, which inhibits the epidermal growth factor receptor, is under study for its potential as a glioma‐targeted therapy combined with nanoparticles [44]. Bexarotene, a third‐generation retinoic acid X receptor agonist primarily used for haematologic malignancies, has also demonstrated potential against glioma cells by inhibiting cell proliferation [45]. Bicalutamide, an androgen receptor antagonist, has been shown to be effective at inhibiting glioma progression both in vivo and in vitro [46]. Bortezomib, a proteasome inhibitor used in treating multiple myeloma, has also shown efficacy in inhibiting glioma growth and enhancing temozolomide chemotherapy [47]. Cisplatin, which is widely used in chemotherapy, has potential in glioma treatment when combined with temozolomide and nanoparticle delivery [48]. Cyclobenzamide, an oncogene inhibitor under investigation for glioma, significantly curtails glioma cell growth when used in combination therapy [49]. Dasatinib, a multikinase inhibitor used in glioma clinical trials, has yet to demonstrate significant efficacy and might require combination therapy [50]. The use of docetaxel and paclitaxel, which are antitumor drugs from the same drug class, for glioma treatment is being investigated, with a focus on nanodrug delivery [51, 52]. Sorafenib, an inaugural oral multikinase inhibitor, effectively induces apoptosis in glioma cells, particularly when used with temozolomide [53]. The efficacy of these chemotherapeutic agents remains under investigation, with studies still in the preclinical or preliminary clinical trial stages, necessitating further research. In this study, we identified chemotherapeutic agents potentially sensitive to glioma patients with varying risk scores, offering potential guidance for clinical application.

In subsequent analyses, using the random forest algorithm, we identified *NSUN6* as the most critical gene in our prognostic model. Data from the Human Protein Atlas (HPA) further confirmed that *NSUN6* expression is elevated in glioma tissues compared to normal brain. *NSUN6* encodes an RNA 5‐methylcytosine (m5C) methyltransferase that modifies both mRNAs and tRNAs [54, 55]. When modifying mRNAs, *NSUN6* promotes translational efficiency and may influence transcript stability indirectly [56]. Although knockout studies in mice revealed no significant changes in mRNA decay, its role in human cancers has attracted increasing attention [57].

Several studies have reported tumor‐suppressive roles of *NSUN6*. Its expression is frequently reduced in pancreatic cancer, and its overexpression significantly inhibits lung cancer development [58, 59]. Conversely, in osteosarcoma, *NSUN6* is upregulated and correlates with poor prognosis, highlighting its context‐dependent role in cancer biology [60]. In gliomas, emerging evidence supports a protective role for *NSUN6*. Awah et al. demonstrated that glioblastoma patients with elevated *NSUN6* expression exhibited better responses to temozolomide chemotherapy and overall prognosis [61]. Similarly, Yang et al. incorporated *NSUN6* expression into a machine learning‐based risk model, where higher expression consistently correlated with lower risk scores and improved survival [62].

In our study, we validated the prognostic significance of *NSUN6* through both bioinformatic and experimental approaches. GSEA revealed that the high *NSUN6* expression was associated with enrichment of tumor‐suppressive pathways, including p53 signaling, E2F target inhibition, mTORC1 regulation, glycolysis and xenobiotic metabolism – all of which are central to glioma cell cycle control, DNA damage response and metabolic adaptation. These results suggest that *NSUN6* may exert its tumor‐suppressive effect through coordinated epigenetic and metabolic regulatory mechanisms.

To experimentally validate its function, we overexpressed *NSUN6* in two glioma cell lines (LN229 and U251) using plasmid transfection. Subsequent phenotypic assays, including CCK‐8 viability tests, colony formation assays and scratch wound healing assays, consistently demonstrated that *NSUN6* overexpression significantly inhibited glioma cell proliferation and migration. These findings indicate that elevated *NSUN6* expression correlates with favorable biological behavior in glioma cells and support its role as a potential tumor suppressor. However, it is important to note that our functional experiments assessed only phenotypic changes. The precise molecular mechanisms by which *NSUN6* modulates these downstream pathways remain to be fully elucidated.

Future studies involving transcriptomic profiling, m5C mapping and pathway perturbation are needed to uncover the specific biological targets and regulatory networks through which *NSUN6* mediates its tumor‐suppressive effects in glioma.

This study has several limitations. First, our research relies primarily on retrospective analyses from multiple glioma databases, which are subject to batch effects, heterogeneous clinical annotations and missing data. In particular, important clinical variables such as the extent of surgical resection and preoperative Karnofsky Performance Status (KPS) were not consistently available across datasets and could not be included in our modelling. Prospective, multicenter clinical studies with comprehensive clinical documentation are required to validate and refine our prognostic model. Second, we have only preliminarily validated the effects of key genes – particularly *NSUN6* – on glioma cells in vitro, without fully elucidating their downstream molecular mechanisms. Additional in vivo and mechanistic studies are required to clarify the functional contributions of these RNA processing factor genes to glioma pathogenesis and therapeutic response.

## 5 Conclusions

In conclusion, we constructed and validated a robust prognostic model based on 19 RNA processing factor genes, which accurately stratifies glioma patients by survival risk across multiple independent datasets. Among these genes, *NSUN6* was identified as a key protective factor, with tumor‐suppressive potential validated by both computational and experimental approaches. The model provides new insights into the molecular heterogeneity of gliomas and holds substantial clinical utility.

By distinguishing high‐risk from low‐risk patients, the model may assist in tailoring treatment intensity – potentially guiding decisions regarding aggressive therapy, inclusion in clinical trials, or conservative management. Moreover, integrating this signature into clinical workflows could improve patient monitoring, facilitate early detection of disease progression and support the development of personalized therapeutic strategies based on individual RNA regulatory landscapes.

## Author Contributions

**Jiarui Chen:** data curation (equal), project administration (equal), visualization (equal), writing – original draft (equal). **Caidi Ying:** data curation (equal). **Zhaowen Gu:** formal analysis (equal), software (equal). **Bingrui Zhu:** data curation (equal), formal analysis (equal), writing – original draft (equal). **Junjie Wang:** data curation (equal), formal analysis (equal). **Yajun Qian:** formal analysis (equal), writing – original draft (equal). **Haiyan Zheng:** funding acquisition (equal), project administration (equal). **Jianming Zhang:** funding acquisition (equal), project administration (equal). **Yongjie Wang:** funding acquisition (equal), investigation (equal), project administration (equal), writing – review and editing (equal).

## Ethics Statement

The authors have nothing to report.

## Consent

The authors have nothing to report.

## Conflicts of Interest

The authors declare no conflicts of interest.

## Supporting information


**Table S1.** Prognostic RNA Processing Factor Genes and Their Coefficients.

## Data Availability

The data that support the findings of this study are available in GEO, TCGA and CGGA. These data were derived from the following resources available in the public domain: https://www.ncbi.nlm.nih.gov/geo/query/acc.cgi; https://portal.gdc.cancer.gov/; https://www.cgga.org.cn/.
