# Supplementary material for: Construction of a Prognostic Model Using RNA Processing Factor Genes and the Key Role of NSUN6 in Glioma Outcomes
Source: J Cell Mol Med. 2025 Jun 25;29(12):e70668. doi: 10.1111/jcmm.70668 (PMC12191409; doi:10.1111/jcmm.70668)
Supplement: Supplementary file 1 — Table S1. Prognostic RNA Processing Factor Genes and Their Coefficients. [file JCMM-29-e70668-s001.docx]

Supplementary Table S1. Prognostic RNA Processing Factor Genes and Their Coefficients

| Gene Symbol | Coefficient | Risk Category | Gene Full Name |
| --- | --- | --- | --- |
| *TRMT11* | -0.015867 | Protective | tRNA Methyltransferase 11 Homolog |
| *CSDC2* | -0.018927 | Protective | Cold Shock Domain Containing C2 |
| *METTL6* | -0.023323 | Protective | Methyltransferase 6, TRNA N3-Cytidine |
| *ECD* | -0.034971 | Protective | Ecdysoneless Cell Cycle Regulator |
| *INTS6* | -0.050674 | Protective | Integrator Complex Subunit 6 |
| *C2orf49* | -0.087581 | Protective | Chromosome 2 Open Reading Frame 49 |
| *HNRNPH2* | -0.108917 | Protective | Heterogeneous Nuclear Ribonucleoprotein H2 |
| *DICER1* | -0.131864 | Protective | Dicer 1, Ribonuclease III |
| *DYRK1A* | -0.183283 | Protective | Dual Specificity Tyrosine Phosphorylation Regulated Kinase 1A |
| *PQBP1* | -0.252425 | Protective | Polyglutamine Binding Protein 1 |
| *MYOD1* | -0.339401 | Protective | Myogenic Differentiation 1 |
| *RCL1* | -0.343328 | Protective | RNA Terminal Phosphate Cyclase-Like 1 |
| *CPEB3* | -0.363275 | Protective | Cytoplasmic Polyadenylation Element Binding Protein 3 |
| *NSUN6* | -0.473516 | Protective | NOP2/Sun RNA Methyltransferase 6 |
| *TRMT2B* | 0.319944 | Risk | tRNA Methyltransferase 2 Homolog B |
| *TYW1* | 0.294518 | Risk | tRNA-YW Synthesizing Protein 1 Homolog |
| *DHX15* | 0.147099 | Risk | DEAH-Box Helicase 15 |
| *KDM1A* | 0.034653 | Risk | Lysine Demethylase 1A |
| *ZFP36L1* | 0.013556 | Risk | ZFP36 Ring Finger Protein Like 1 |
